# Lysine-PEGylated Cytochrome C with Enhanced Shelf-Life Stability

**DOI:** 10.3390/bios12020094

**Published:** 2022-02-04

**Authors:** João H. P. M. Santos, Valker A. Feitosa, Giovanna P. Meneguetti, Gustavo Carretero, João A. P. Coutinho, Sónia P. M. Ventura, Carlota O. Rangel-Yagui

**Affiliations:** 1 Department of Biochemical and Pharmaceutical Technology, School of Pharmaceutical Science, University of São Paulo, São Paulo 05508-000, Brazil; valker@usp.br (V.A.F.); gimeneguetti@ipt.br (G.P.M.); 2 Bionanomanufacturing Center, Institute for Technological Research, São Paulo 05508-901, Brazil; 3 Department of Biochemistry, Institute of Chemistry, University of São Paulo, São Paulo 05508-000, Brazil; gustavo.carretero@usp.br; 4 Department of Chemistry, CICECO—Aveiro Institute of Materials, University of Aveiro, 3810-193 Aveiro, Portugal; jcoutinho@ua.pt (J.A.P.C.); spventura@ua.pt (S.P.M.V.)

**Keywords:** bioconjugation, cytochrome-c, lysine PEGylation, long-term stability

## Abstract

Cytochrome c (Cyt-c), a small mitochondrial electron transport heme protein, has been employed in bioelectrochemical and therapeutic applications. However, its potential as both a biosensor and anticancer drug is significantly impaired due to poor long-term and thermal stability. To overcome these drawbacks, we developed a site-specific PEGylation protocol for Cyt-c. The PEG derivative used was a 5 kDa mPEG-NHS, and a site-directed PEGylation at the lysine amino-acids was performed. The effects of the pH of the reaction media, molar ratio (Cyt-c:mPEG-NHS) and reaction time were evaluated. The best conditions were defined as pH 7, 1:25 Cyt-c:mPEG-NHS and 15 min reaction time, resulting in PEGylation yield of 45% for Cyt-c-PEG-4 and 34% for Cyt-c-PEG-8 (PEGylated cytochrome c with 4 and 8 PEG molecules, respectively). Circular dichroism spectra demonstrated that PEGylation did not cause significant changes to the secondary and tertiary structures of the Cyt-c. The long-term stability of native and PEGylated Cyt-c forms was also investigated in terms of peroxidative activity. The results demonstrated that both Cyt-c-PEG-4 and Cyt-c-PEG-8 were more stable, presenting higher half-life than unPEGylated protein. In particular, Cyt-c-PEG-8 presented great potential for biomedical applications, since it retained 30–40% more residual activity than Cyt-c over 60-days of storage, at both studied temperatures of 4 °C and 25 °C.

## 1. Introduction

Cytochrome c (Cyt-c) is a small protein with 104 amino acids and a molecular weight of about 12 kDa. It is a heme protein involved in mitochondrial electron transfer, even though it is not considered a natural enzyme; it catalyzes several chemical reactions, including hydrogen peroxide reduction, aromatic oxidation, hydroxylation, epoxidation and *N*-demethylation [1]. Based on the broad heterogeneity obtained in biotransformation reactions catalyzed by Cyt-c, along with its high reactivity towards different substrates and its electron transfer capability, this protein has been recently explored as a biosensor of hydrogen peroxide, nitric oxide and polycyclic aromatic hydrocarbons [1,2,3]. Furthermore, Cyt-c has been studied as an anticancer biopharmaceutical candidate in nanosized, cancer-cell targeted delivery systems [4,5,6,7,8]. However, the potential biosensing and biopharmaceutical applications of Cyt-c are compromised by irreversible denaturation, aggregation and/or precipitation. Its instability results from the fragile structure shared by most proteins.

The stability of biosensors during storage is crucial to guarantee reproducibility and feasibility of biochemical measurements. Generally, long-term stability of protein-based sensors is the major obstacle to their potential application [9]. Along the same line, long-term stability of biopharmaceuticals is extremely important (i.e., it is one of the crucial criteria for biopharmaceutical development). In this sense, chemical modifications of proteins are a key solution to overcome these drawbacks. PEGylation is one of the most common chemical modifications applied in the protein-based biosensing field and in the design of novel, improved and more stable biopharmaceuticals, mostly due to the advantages it may confer to proteins [2].

The chemical bonding of polyethylene glycol (PEG) to the protein increases the protein’s solubility in water and some organic solvents (such as dichloromethane), and provides long-term stability while reducing denaturation, aggregation and precipitation, as well as preventing immunogenicity and proteolytic degradation due to the PEG shielding effect [10,11,12,13]. Other bioconjugation techniques to improve stability of proteins are PASylation, XTEN, fusion proteins and glycosylation. The literature shows that many redox proteins with sensing applications have already been PEGylated to improve stability, e.g., horseradish peroxidase [14], hemoglobin [15] and myoglobin [16].

Recent studies showed that PEGylation could kinetically stabilize the Cyt-c structure through maintenance of the heme group, while decreasing the unfolding rate [2,17]. However, most of the PEGylation reactions reported in the literature in the field of biosensors are not site-specific, resulting in the random production of PEGylated conjugates with increased polydispersity [3,18]. This unspecific reaction leads to decreased bioactivity and the lack of batch-to-batch control, resulting in a mixture of protein-based biosensors with differential biosensing activity [10,19]. To overcome this problem, we developed a site-specific PEGylation protocol for Cyt-c through the conjugation of methoxy PEG succinimidyl NHS ester (mPEG-NHS). This functionalized mPEG is attached to the protein through a nucleophilic attack of primary protein amine groups, forming an ester bond and releasing an NHS group. Reaction conditions, such as pH, molar proportion of Cyt-c:PEG and reaction time, were studied, and the effect of PEGylation on Cyt-c chemical structure, function and stability over time was examined. The results pointed to Cyt-c-PEG-8 (with eight mPEG molecules attached) as the conjugate with the highest stability, showing that this chemical modification actual preserves the protein structure and function. 

## 2. Materials and Methods

### 2.1. Materials

Horse heart cytochrome c (Cyt-c, ≥95% purity), 2,2′-azino-di-(3-ethylbenzthiazoline sulfonic acid) (ABTS, ≥95% purity), hydrogen peroxide (99% purity) and hydroxylammonium chloride (99% purity) were obtained from Sigma-Aldrich^®^ (St. Louis, MO, USA). Methoxy PEG *N*-hydroxysuccinimide ester (mPEG-NHS, 5 kDa) with high purity was acquired from Nanocs^®^ (New York, NY, USA). The aqueous buffer used in the PEGylation reaction was potassium phosphate buffer (100 mM). All other reagents were of analytical grade. The water used was double-distilled, passed through a reverse osmosis system and was further treated with Direct-Q^®^ 8 UV remote water purification system (Merck^®^, São Paulo, Brazil).

### 2.2. Cyt-c PEGylation Reaction

**Figure 1:**
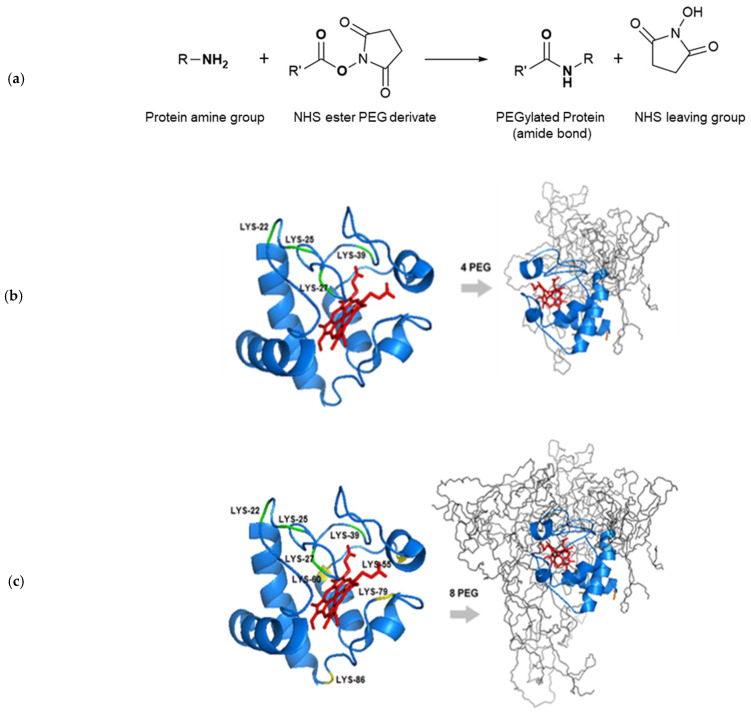
Representation of PEGylation reaction of cytochrome c (Cyt-c) for the attachment of 4 and 8 PEG molecules. (**a**) Schematic overview of PEGylation reaction in primary amine groups of Cyt-c with methoxy polyethylene glycol *N*-hydroxysuccinimide (mPEG-NHS). Graphical representation of theoretical lysine residues (Lys) on Cyt-c (PDB code 1HRC) and its (**b**) 4-PEGylated, and (**c**) 8-PEGylated counterparts using the PyMOL^®^ software. Gray lines in the Cyt-c PEGylated forms represent attached mPEG chains.

The PEGylation reaction was carried following the method provided by the PEG derivative supplier [20]. A schematic description of the PEGylation reaction can be found in Figure 1a. Briefly, Cyt-c was dissolved in 0.1 M of potassium phosphate buffer and allowed to react with mPEG-NHS at room temperature and constant magnetic stirring at 400 rpm. Several parameters were optimized, namely the pH (range from 7 to 12), protein:mPEG-NHS molar proportion (1:5, 1:10, 1:25 or 1:35) reaction time (15, 30 or 45 min). At the end, 2 M of hydroxylamine (1:10 *v*/*v*) was added to stop the reaction as well as to avoid the production of undesirable and unstable products (such as ester conjugation). 

### 2.3. In Silico Studies: Molecular Visualization of Cyt-c PEGylation

The pK_a_ of *N*-terminal and lysine residues was determined using the software H++ version 3.2 (Virginia Tech, Blacksburg, VA, USA) to understand the possible sites of mPEG attachment in Cyt-c (crystal structure PDB code: 1HRC) [21]. Additionally, molecular models of Cyt-c were constructed using PyMOL version 1.3 (Molecular Graphics System^®^, Schrödinger, LLC, New York, NY, USA) based on the Cyt-c crystal structure. In silico models were generated of four and eight molecules of mPEG (5 kDa) attached to lysine residues of Cyt-c to form Cyt-c-PEG-4 (Lys 22, 25, 27 and 39) and Cyt-c-PEG-8 (Lys 22, 25, 27, 39, 55, 60, 79 and 86), respectively. All mPEG chains were auto-sculpted to adjust molecular conformation.

### 2.4. Purification of PEGylated Conjugates

PEGylation reaction mixtures were purified by size exclusion chromatography (SEC) using a Superdex™ 200 Increase 10/300 GL column (crosslinked agarose–dextran resin) (Cytiva^®^, Marlborough, MA, USA) in an AKTA™ purifier system (Cytiva^®^, Marlborough, MA, USA) [22]. The column was equilibrated with 0.01 M phosphate buffer (0.14 M NaCl, pH 7.4) and eluted with the same buffer at a flow of 0.75 mL.min^−1^. The protein fractions (determined by UV at 280 nm) corresponding to unreacted Cyt-c and modified proteins Cyt-c-PEG-4 (i.e., protein with 4 mPEG molecules attached) and Cyt-c-PEG-8 (i.e., protein with 8 mPEG molecules attached) were stored at −20 °C for further study. Cyt-c concentration was determined based on a calibration curve established in the SEC-FPLC at the conditions described before. The retention time of Cyt-c (confirmed with the commercial and pure sample) was found to be ca. 24 min within an analysis time of 40 min. The percentage yield of native and modified Cyt-c conjugates was calculated by dividing the FPLC peak area corresponding to the target protein by the total area of all peaks corresponding to the native protein and all conjugates present in the sample.

### 2.5. Determination of PEGylation Degree of Cyt-c Conjugates by SEC

The PEGylation degree of the Cyt-c conjugates was determined by SEC [22,23]. The molecular weight (MW) of protein conjugates and, consequently, the degree of PEGylation was determined by column (Superdex™ 200 Increase 10/300 GL, Cytiva^®^, Marlborough, MA, USA) calibration with several proteins of known molecular weight, and the void volume (V_0_) was determined using blue dextran 2000. All standards were from Cytiva^®^, Marlborough, MA, USA. The standard proteins were run at the same conditions assayed for the SEC purification of Cyt-c conjugates: 0.01 M phosphate buffer (with 0.14 M NaCl, pH 7.4), at 0.75 mL·min^−1^ flow rate. The MW of the protein conjugates was determined by the linear relationship obtained by plotting the *K_av_* value of the proteins (as calculated by Equation (1)) and the logarithms of their MW. The calibration curve is presented in Supplementary Materials Figure S1.

(1) $$K_{av}=\frac{\left(V_{e}-V_{0}\right)}{\left(V_{T}-V_{0}\right)}$$

where elution volume (*V_e_*) is the amount of eluent collected from the start of loading the sample to the point of its maximal elution; (*V_T_*) corresponds to the total volume of the column.

The MW determined by this technique with globular proteins seems to be accurate to about ±10%, resulting in the uncertainty in the average number of PEGs per Cyt-c to be less than ±1. Chromatograms corresponding to each specific PEGylation condition are presented in the Supplementary Materials (Figures S2, S4 and S6).

### 2.6. Gel Electrophoresis

Protein samples with 20 µL of volume were applied to a 12% polyacrylamide gel (10 cm × 10.5 cm, 0.75 mm thick) under reducing conditions (SDS-PAGE) according to Laemmli (1970) [24]. The electrophoretic run was performed by applying a 120 V potential for approximately 1 h 30 min using a vertical Mini Protean™ system (Bio-Rad^®^, Hercules, CA, USA). Protein marker Precision Plus Protein (#1610374, Bio-Rad^®^, Hercules, CA, USA) was used. Gels were stained with Coomassie Brilliant Blue R-250 (Thermo Scientific^®^, Waltham, MA, USA) for visualization of protein bands. 

### 2.7. Circular Dichroism (CD) Spectroscopy

CD spectra of Cyt-c and its modified forms were obtained in a J-720 Spectropolarimeter (Jasco^®^, Tokyo, Japan). The final spectra were the average of six scans, with subtraction of the buffer spectrum (0.01 M phosphate buffer, 0.14 M NaCl, pH 7.4). CD spectra were obtained in far UV (190–260 nm). Samples were placed in 1 mm-optical-length quartz cells, with concentration ranging from 6 to 15 µM. Spectra intensities (*θ*, mdeg) were converted to delta epsilon (Δε, cm^−1^·M^−1^) based on Equation (2):

(2) $$\Delta \varepsilon =\frac{\theta }{32.98\;.\;C\;.l}$$

where (*C*) is the protein concentration in mol·L^−1^, and (*l*) is the optical length in cm.

### 2.8. Determination of Cyt-c Long-Term Stability

Long-term stability was investigated at 4 °C (refrigerated) and room temperature (~25 °C). The peroxidase activity was measured across 60 days and the residual activity (%) calculated, with the activity on day 1 considered 100%. The enzyme-like activity of Cyt-c was determined by the catalytic oxidation of 50 µM ABTS in the presence of 0.5 mM hydrogen peroxide [25]. The concentration of Cyt-c or PEGylated Cyt-c forms was 10 µM in 0.01 M phosphate buffer (with the addition of 0.14 M NaCl, pH 7.4). The reaction was initiated by the addition of hydrogen peroxide, and the increase in absorbance at 418 nm was measured in a SpectraMax Plus 384 (Molecular Devices^®^, California, CA, USA) spectrophotometer. 

## 3. Results and Discussion

### 3.1. Cyt-c PEGylation

Regarding the design of site-specific polymer-protein conjugates, the selection of appropriated conditions for the PEGylation reaction is the first step towards obtaining a successful site-specific PEGylated product. Several final properties of the conjugate are directly related to the physicochemical properties of the selected PEG derivative [12,26,27]. Moreover, the number and local reactivity of available attachment sites in the amino acid sequence of the target protein are important criteria to choose the type of PEGylation reaction [26]. Therefore, the reaction design must be tailored to the protein of interest, depending on its physicochemical properties, amino acid sequence and final application [11,28]. Taking this into account, the amino (-NH_2_) reactive PEGylation, using *N*-hydroxysuccinimide (NHS) functionalized methoxy polyethylene glycol (mPEG-NHS), was selected due to its inherent advantages. In this kind of PEGylation, the PEG polymer is generally attached to the ɛ amino group of lysine by a nucleophilic attack of the amine group to the carboxyl of the reactive mPEG, releasing NHS as a byproduct (Figure 1a) [29,30]. Compared to other mPEG-NHS ester derivatives, succinimidyl carbonate-functionalized mPEG-NHS offers superior reactivity and higher stability in aqueous solution [20].

### 3.2. Formatting of Mathematical Components

The PEGylation of Cyt-c with mPEG-NHS resulted in three different degrees of polymer conjugation: representing the attachment of four mPEG molecules (Cyt-c-PEG-4), eight mPEG molecules (Cyt-c-PEG-8) and a poly PEGylated form (Cyt-c-Poly PEG). In order to understand the location of PEGylation sites in Cyt-c, bioinformatics studies were conducted to determine the pK_a_ of the amino acids that can be PEGylated (Table 1) and to construct the molecular models of Cyt-c-PEG-4 (Figure 1b) and Cyt-c-PEG-8 (Figure 1c).

Primary amines exist at the *N*-terminus of each polypeptide chain and in the side-chain of lysine (Lys, K). PEGylation of amine groups preferentially takes place in deprotonated primary amines, in which the lone pair of electrons is freely available for the nucleophilic attack; therefore, it depends on the residue’s pK_a_. The location in the polypeptide chain is also important, since the amino acids more exposed to the solvent and located in flexible regions of the protein are more likely to be PEGylated.

As shown in Table 1, Cyt-c contains one *N*-terminal residue (Met) and 19 Lys residues that can be potentially modified by mPEG covalent binding. Of these, six are part of loop regions (four in the largest loop more accessible to mPEG and two in an intermediate loop), three are in the alpha-helix transition, and the remaining (10) are located in the alpha-helix composition. According to previous studies, the lysine residues more available for mPEG attachment are Lys22, Lys25, Lys27 and Lys39, all located in the flexible region (Ω loop) of the Cyt-c polypeptide chain [18]. In this sense, the site-specific PEGylated form Cyt-c-PEG-4 refers to mPEG attachment to those amino acid residues (Figure 1b). Regarding the other four lysine residues covalently bound to mPEG in Cyt-c-PEG-8, Lys55, Lys60, Lys79 and Lys86 are the most probable binding sites (Figure 1c). These lysine residues are present in flexible regions of the protein (Ω loop and alpha-helix transition). The *N*-terminal group of Cyt-c is less prone to PEGylation at neutral pH due to the extremely high pK_a_, which makes it preferentially protonated at most pH values, therefore hampering the nucleophilic attack.

### 3.3. Effect of pH on the PEGylation of Cyt-c

PEGylation reactions are mostly conducted in single-step unidirectional batch systems to guarantee that all products have followed the same procedure, enhancing validation, reproducibility and optimization of the reaction. One of the key parameters to design a site-specific PEGylation reaction at amino groups is the pH. By controlling pH, PEGylation can be manipulated to produce specific conjugates, an essential feature to promote batch-to-batch control. 

**Figure 2:**
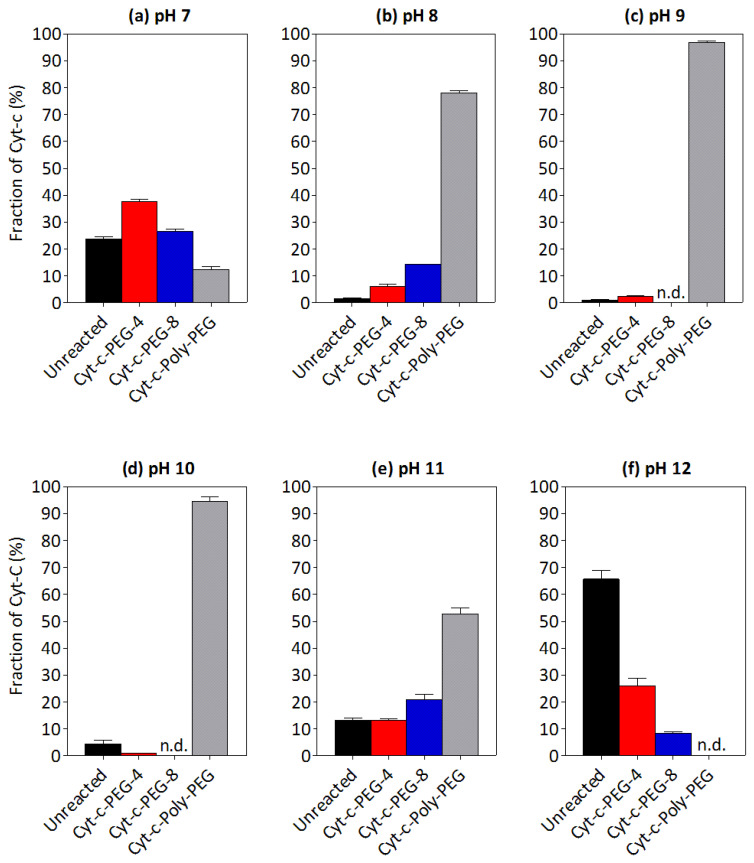
Effect of pH on cytochrome c (Cyt-c) PEGylation yield (%) at different pH values: (**a**) pH = 7, (**b**) pH = 8, (**c**) pH = 9, (**d**) pH = 10, (**e**) pH = 11 and (**f**) pH = 12. The PEGylation reaction was performed in 0.1 M potassium phosphate buffer, 1:25 molar proportion (Cyt-c:mPEG-NHS, 5 kDa), during 30 min: n.d., not detected.

The effect of pH on Cyt-c PEGylation is presented in Figure 2. As can be seen, the number of PEGylated forms increases with the pH due to the higher probability of lysine residues to react with mPEG derivative, since in pH > pK_a_ they are deprotonated. The native protein amount is also lower in more alkaline pH, i.e., from pH 7 (Figure 2a) to pH 9 (Figure 2c), probably as a result of a higher degree of PEGylation. However, the opposite trend is observed for pH > 10: the degree of PEGylation decreases and the concentration of native protein increases to 66% at pH 12 (Figure 2f). This behavior can be explained by the higher hydrolysis rate of the mPEG-NHS in alkaline solutions, leading to lower concentrations of this reagent and, consequently, decreasing PEGylation yield. The highest yields of Cyt-c-PEG-4 (38%) and Cyt-c-PEG-8 (26%) were both observed at pH 7 (Figure 2a), defined as the optimal pH in this specific case.

Figures S2 and S3, respectively, show the chromatograms and electrophoretic profiles (SDS-PAGE) of Cyt-c PEGylation reaction media at different pH values. The results depicted in Figure S3 seem to corroborate the PEGylation yield data (Figure 2), with each band representing one, site-specific Cyt-c-PEGylated form as shown by the peaks of the chromatograms (Figure S2). The bands presenting higher intensity correspond to pH 7–12, as opposed to those obtained for the pH range between 8 and 10, in which the most intense bands correspond to the poly PEGylated forms.

Although the effect of pH on the PEGylation of Cyt-c is practically unexplored, this parameter was revealed to be extremely important in the manipulation of a site-specific reaction. The influence of pH on protein PEGylation has been already studied for other proteins, such as lysozyme [31], horseradish peroxidase (HRP) [32], *N*-carbamoyl-L-amino acid amidohydrolase (L-*N*-carbamoylase) [32] and rh-interferon-α2B [33], in which the important effect of this parameter was also observed. In these studies, the rate of PEGylation was found to increase with pH, in agreement with the results obtained for Cyt-c PEGylation. Nonetheless, only a narrow range of pH (i.e., from 6 to 8) was evaluated in the literature. Thus, this study enlarges the knowledge of the effect of pH on protein PEGylation.

### 3.4. Effect of mPEG Derivative Concentration on the Cyt-c PEGylation Reaction

**Figure 3:**
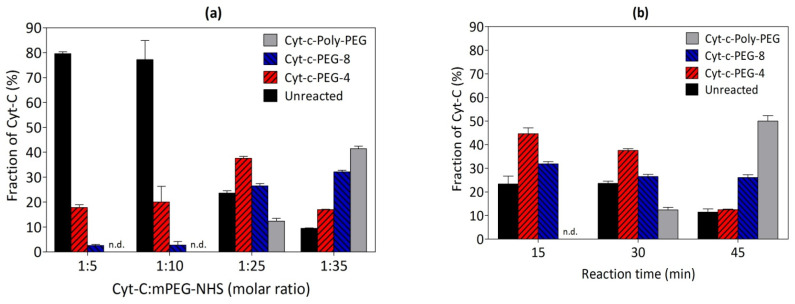
(**a**) Effect of protein:mPEG-NHS molar ratio on cytochrome c (Cyt-c) PEGylation yield (%). The PEGylation reaction was performed at pH 7 for 30 min. (**b**) Effect of reaction time on Cyt-c PEGylation yield. The PEGylation reaction was performed in 0.1 M potassium phosphate buffer (pH 7), 1:25 molar proportion (protein:mPEG-NHS): n.d., not detected.

The mPEG derivative concentration is an important parameter and influences reaction yields. The mPEG derivative (mPEG-NHS) was investigated at 1:5, 1:10, 1:25 and 1:35 Cyt-c:mPEG-NHS molar ratios, and results are presented in Figure 3a, while Figures S4 and S5, respectively, correspond to the resultant chromatograms and representative SDS-PAGE of the reaction media.

The lowest molar ratio values, specifically 1:5 and 1:10, resulted in lower PEGylation yields and higher amounts of unreacted Cyt-c present. In addition, the poly PEGylated forms were not detected at those experimental conditions (Figure 3a). The molar ratio 1:35 resulted in polydispersity of the polyPEGylated products due to the excess of mPEG and, therefore, polydispersity [18,32]. In this sense, the optimal result was obtained at molar ratio 1:25, which corresponded to higher yield of site-specific Cyt-c-PEGylated forms and, as already discussed, less unreacted protein.

### 3.5. Effect of Reaction Time on the PEGylation of Cyt-c

The reaction time for Cyt-c PEGylation was also investigated for 15, 30 and 45 min (Figure 3b). According to the results, the poly PEGylation yield increases with the reaction time. Therefore, higher yield of Cyt-c-PEG-4 (45%) and Cyt-c-PEG-8 (32%) were obtained in the shortest time of 15 min, with no poly PEGylated forms produced, which could facilitate the purification process. Figures S6 and S7 show the chromatograms and SDS-PAGE, respectively, of Cyt-c PEGylation reaction at the studied times. The effect of the reaction time on Cyt-c PEGylation yield was already described in the literature [18], in which the PEGylation yield and the polydispersity increased with time. Actually, the shorter time, which was selected as the best condition in this work, represents an economic improvement of the overall process, when compared, for example, with the two hours (with a total PEGylation yield of 60.2 ± 0.7%) defined in the literature [18] as the best condition.

**Figure 4:**
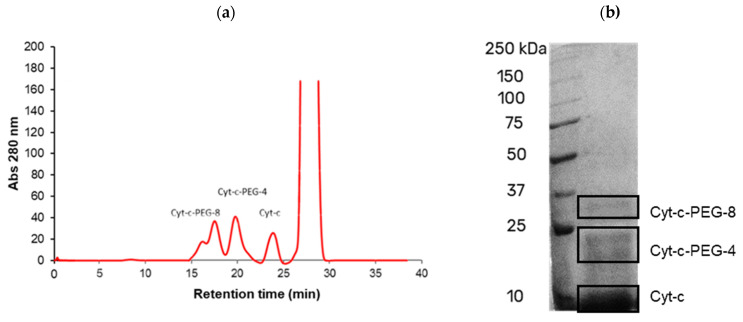
Chromatogram (**a**) and electrophoretic profile (**b**) of cytochrome c (Cyt-c) after PEGylation reaction under best experimental conditions: pH 7, 1:25 molar ratio (Cyt-c:mPEG-NHS) and reaction time 30 min: Cyt-c-PEG-4, 4 mPEG molecules attached; Cyt-c-PEG-8, 8 mPEG molecules attached.

Summing up, a designed and tailor-made PEGylation reaction was developed, and the best experimental conditions were determined to be pH 7, 1:25 molar ratio (Cyt-c:mPEG-NHS) and reaction time of 15 min. At these conditions, two forms of site-specific PEGylated Cyt-c (Cyt-c-PEG-4 and Cyt-c-PEG-8) were obtained with no undesirable poly PEGylated forms identified by chromatogram (Figure 4a) and SDS-PAGE (Figure 4b). These two PEGylated Cyt-c forms were purified through SEC-FPLC and further applied, as saline solution (sodium phosphate buffer, 0.14 M NaCl, pH 7.4), in spectroscopic studies to understand the effect of PEGylation on the Cyt-c structure, as well as on the long-term stability of the conjugates obtained, as shown below.

### 3.6. Effect of PEGylation on the Cyt-c Structure

**Figure 5:**
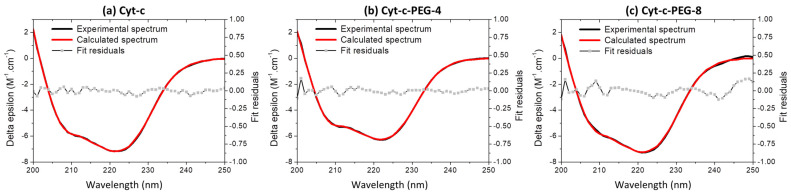
Experimental far-UV CD spectra (black lines) of Cyt-c (**a**), Cyt-c-PEG-4 (**b**), and Cyt-c-PEG-8 (**c**) in 0.01 M sodium phosphate buffer (0.14 M NaCl, pH 7.4); sample concentration ranged from 6 to 15 µM [34]. Theoretical CD spectra (red lines) of native and PEGylated Cyt-c conjugates calculated from experimental spectra using BestSel algorithm, and fit residuals.

To understand the effect of PEGylation on Cyt-c secondary and tertiary structures, CD measurements have already been performed, as previously shown by the authors of [34]. CD spectra of native and PEGylated Cyt-c present negative bands centered at 222 and 208 nm, characteristics of a high content of α-helical secondary structure (Figure 5), in agreement with the horse heart Cyt-c high-resolution three-dimensional structure solved by X-ray diffraction crystallography [21]. As already shown by other authors [2,17], the main results obtained in this work also show that PEGylation reaction of Cyt-c caused no significant spectral changes, proving that the protein secondary structure is maintained. CD data were further analyzed by spectra deconvolution using BeStSel online software [35] and confirmed that the PEGylated forms kept their secondary structure content through the PEGylation reaction and protein purification steps (Figure 5, Table 2). The predominant α-helical secondary structure is maintained in the protein conjugates, with small deviations in the 8x-PEG form (Table 2).

### 3.7. Effect of PEGylation on Long-Term Stability

**Figure 6:**
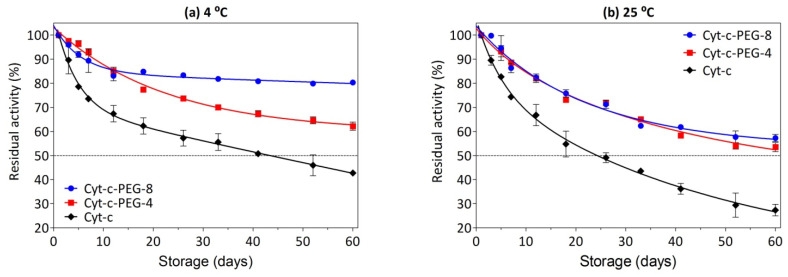
Stability of native and PEGylated forms of cytochrome c (Cyt-c) stored at 4 °C (**a**) and 25 °C (**b**). Residual peroxidative-like activity of Cyt-c (control) was determined by the catalytic oxidation of 50 µM ABTS in the presence of 0.5 mM hydrogen peroxide. The concentration of native and PEGylated Cyt-c forms was 10 µM in 0.01 M phosphate buffer (0.14 M NaCl, pH 7.4).

Considering the further application of Cyt-c and PEGylated forms as either biosensors or anticancer biopharmaceutical, it is necessary to demonstrate structural stability. Therefore, the residual Cyt-c activity was measured by considering the ABTS oxidation by hydrogen peroxidase after long-term storage at different temperatures. The results show that both native Cyt-c and PEGylated Cyt-c forms in saline solution retained the peroxidase activity for several days of storage at both refrigerated (4 °C) and room (25 °C) temperatures (Figure 6a and Figure 6b, respectively). As summarized in Table S1, unPEGylated Cyt-c presents a half-life of approximately 44 days at 4 °C and approximately 24 days at room temperature (~25 °C), while Cyt-c-PEG-8 and Cyt-c-PEG-4 present half-life beyond 60 days at both temperatures, hence leading to an improvement in shelf stability.

Cyt-c-PEG-4 and Cyt-c-PEG-8 showed higher residual activity than the native protein. When stored up to 60 days at 4 °C, Cyt-c-PEG-8 and Cyt-c-PEG-4 retained 80% and 62% of their activity, respectively, while Cyt-c retained only 43% of its activity (Figure 6a and Table S1). As expected, Cyt-c (unPEGylated and PEGylated forms) was more stable at the lower temperature. At room temperature (~25 °C) Cyt-c residual activity was only 27%, contrasting with ≥53% for both PEGylated forms (Figure 6b and Table S1). Indeed, a common characteristic of a large number of enzymes is their low shelf-stability at ambient temperature, especially in solution, owing to protein aggregation, precipitation or even hydrolysis [36,37]. The Cyt-c with higher mPEG content (i.e., Cyt-c-PEG-8) presented superior stability compared to Cyt-c-PEG-4, particularly when under refrigeration. Furthermore, a previous study from our group disclosed an additional benefit of PEGylation regarding its thermoprotective role on Cyt-c, especially at higher temperatures (i.e., 70–95 °C). The enzyme half-life (t_1/2_), which is a key parameter in terms of economic feasibility and resistance to thermal inactivation, was constantly superior in the studied temperatures for PEGylated Cyt-c compared to native Cyt-c, with a more pronounced thermostability for Cyt-c-PEG-8 (i.e., the conjugate with the higher PEGylation degree) [36].

These results demonstrate that site-specific PEGylation is a valuable strategy to increase Cyt-c stability and shelf life. In general terms, it could be a helpful approach to increase the stability of dried proteins (e.g., lyophilized form) [38] or proteins immobilized in solid matrixes [39]. Other works from the literature present alternative strategies to improve long-term stability of Cyt-c, such as storage in an aqueous solution of cholinium-based ionic liquids [40], aminoacid-based ionic liquids [41], surface active ionic liquids [42,43] and deep eutectic solvents (e.g., choline chloride ([Ch]Cl) and ethylammonium chloride (EAC)) [44]. However, these alternative solvents, such as deep eutectic solvents and ionic liquids, are not yet approved in pharmaceutical formulations. For that reason, polishing steps would be necessary to completely remove the solvent of a protein formulation. PEGylation, on the other hand, not only proved to be efficient in stabilizing Cyt-c for extended times, but also is a technology already approved by the FDA for other proteins. Overall, site-specific PEGylation could be associated with biosensing and therapeutical formulations to further improve Cyt-c long-term stability and its potential to be applied as a biopharmaceutical.

## 4. Conclusions

In this work, Cyt-c PEGylated conjugates, namely Cyt-c-PEG-4 and Cyt-c-PEG-8, were produced. The optimal PEGylation conditions for site-specific reaction were found to be pH 7, 1:25 of molar ratio (Cyt-c:mPEG-NHS) and 15 min of reaction, resulting in a yield of 45% for Cyt-c-PEG-4 and 32% for Cyt-c-PEG-8. Moreover, circular dichroism analysis proved that the PEGylation process did not result in significant structural changes in Cyt-c. Additionally, Cyt-c-PEGylated forms were found to be more stable over time than the native protein, with higher stability observed for Cyt-c-PEG-8. Therefore, Cyt-c PEGylation clearly brings advantages through the possibility of extending product shelf-life.

## Figures and Tables

**Table 1:** pK_a_ and location of the lysine (Lys) residues and *N*-terminal moiety of cytochrome c (PDB code 1HRC code).

Residue	Theoretical pK_a_	Location/Secondary Structure
*N* terminal	11.8	*N* terminal moiety
Lys5	>12.0	alpha-helix
Lys7	10.9	alpha-helix
Lys8	9.7	alpha-helix
Lys13	10.8	alpha-helix
Lys22	9.9	Ω loop
Lys25	11.2	Ω loop
Lys27	10.9	Ω loop
Lys39	10.3	Ω loop
Lys53	10.7	alpha-helix
Lys55	10.4	alpha-helix transition
Lys60	10.4	alpha-helix transition
Lys72	8.6	alpha-helix
Lys73	11.0	alpha-helix
Lys79	10.4	Ω loop
Lys86	10.3	Ω loop
Lys87	10.7	alpha-helix transition
Lys88	10.4	alpha-helix
Lys99	>12.0	alpha-helix
Lys100	10.7	alpha-helix

**Table 2:** Secondary structure of native and PEGylated Cyt-c calculated from CD spectra of the enzyme [34].

Cyt-c Form	Alpha	Beta	Random
Native	41	22	37
Cyt-c-PEG-4	41	19	40
Cyt-c-PEG-8	38	26	36

## Data Availability

Not applicable.

## Supplementary Materials

The following are available online at https://www.mdpi.com/article/10.3390/bios12020094/s1, Figure S1: Calibration curve of size exclusion chromatography (SEC), Figure S2: Effect of pH on cytochrome c (Cyt-c) PEGylation yield, Figure S3: SDS-PAGE for the conjugation of mPEG-NHS to cytochrome c (Cyt-c) at different pHs, Figure S4: Effect of molar ratio (protein:mPEG-NHS) on cytochrome c (Cyt-c) PEGylation yield, Figure S5: SDS-PAGE for the conjugation of mPEG-NHS with cytochrome c (Cyt-c) at different molar ratios (protein:mPEG-NHS), Figure S6: Effect of reaction time on cytochrome c (Cyt-c) PEGylation yield, Figure S7: SDS-PAGE for the conjugation of mPEG-NHS with cytochrome c (Cyt-c) for different reaction times, Table S1: Half-life (t_1/2_) and long-term residual activity of native and PEGylated forms of cytochrome c (Cyt-c) stored in phosphate buffer at 4 °C and 25 °C.
