# Supplementary material for: Lysine-PEGylated Cytochrome C with Enhanced Shelf-Life Stability
Source: Biosensors (Basel). 2022 Feb 4;12(2):94. doi: 10.3390/bios12020094 (PMC8869816; doi:10.3390/bios12020094)
Supplement: Supplementary file 1 [file biosensors-12-00094-s001.zip › biosensors-1561193-supplementary.pdf]

Supplementary Materials

Lysine-PEGylated Cytochrome c with Enhanced Shelf-Life Stability

João H. P. M. Santos[1,2*], Valker A. Feitosa[1,2], Giovanna P. Meneguetti[1,2], Gustavo Carretero[3], João A. P. Coutinho[4], Sónia P. M. Ventura[4] and Carlota O. Rangel-Yagui[1*]

1 Department of Biochemical and Pharmaceutical Technology, School of Pharmaceutical Science, University of São Paulo, São Paulo 05508-000, Brazil; valker@usp.br (V.A.F.); gimeneguetti@ipt.br (G.P.M.)
2 Bionanomanufacturing Center, Institute for Technological Research, São Paulo 05508-901, Brazil
3 Department of Biochemistry, Institute of Chemistry, University of São Paulo, São Paulo 05508-000, Brazil; gustavo.carretero@usp.br
4 CICECO—Aveiro Institute of Materials, Department of Chemistry, University of Aveiro, 3810-193 Aveiro, Portugal; jcoutinho@ua.pt (J.A.P.C.); spventura@ua.pt (S.P.M.V.)
* Correspondence: jhsantos@usp.br (J.H.P.M.S.); corangel@usp.br (C.O.R.Y.)

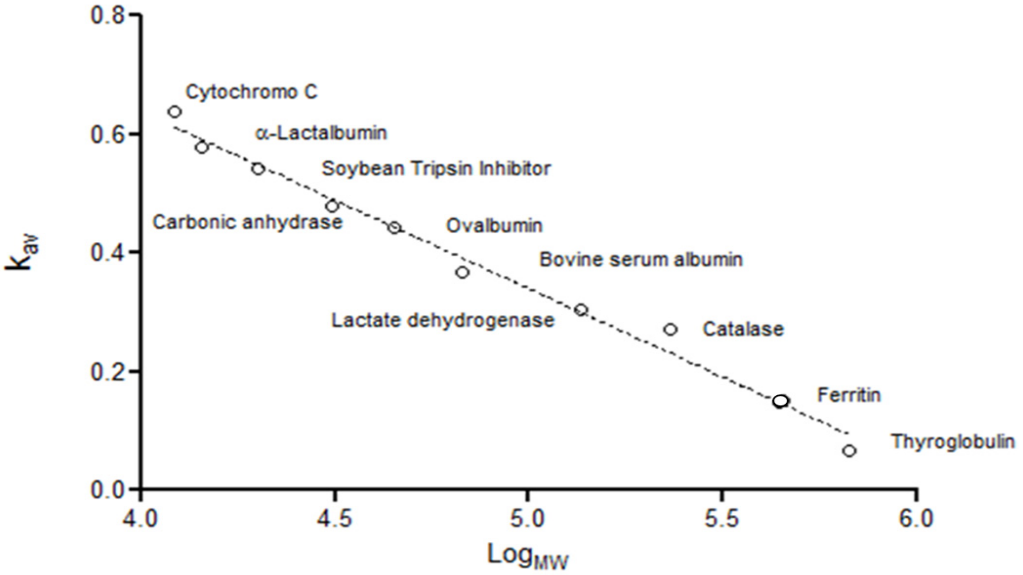

Figure S1. Calibration curve of size exclusion chromatography (SEC) using a Superdex 200 Increase 10/300 GL column (crosslinked agarose-dextran resin, Cytiva) in an AKTA™ purifier system (Cytiva). Data obtained by plotting the Kav value of each protein marker and the logarithms of their molecular weights (MW), namely: thyroglobulin 669 kDa, ferritin 450 kDa, catalase 232 kDa, lactate dehydrogenase 136.7 kDa, bovine serum albumin 67 kDa, ovalbumin 45 kDa, carbonic anhydrase 31 kDa, soybean trypsin inhibitor 20.1 kDa, α-lactalbumin 14.4 kDa and cytochrome c 12.2 kDa.

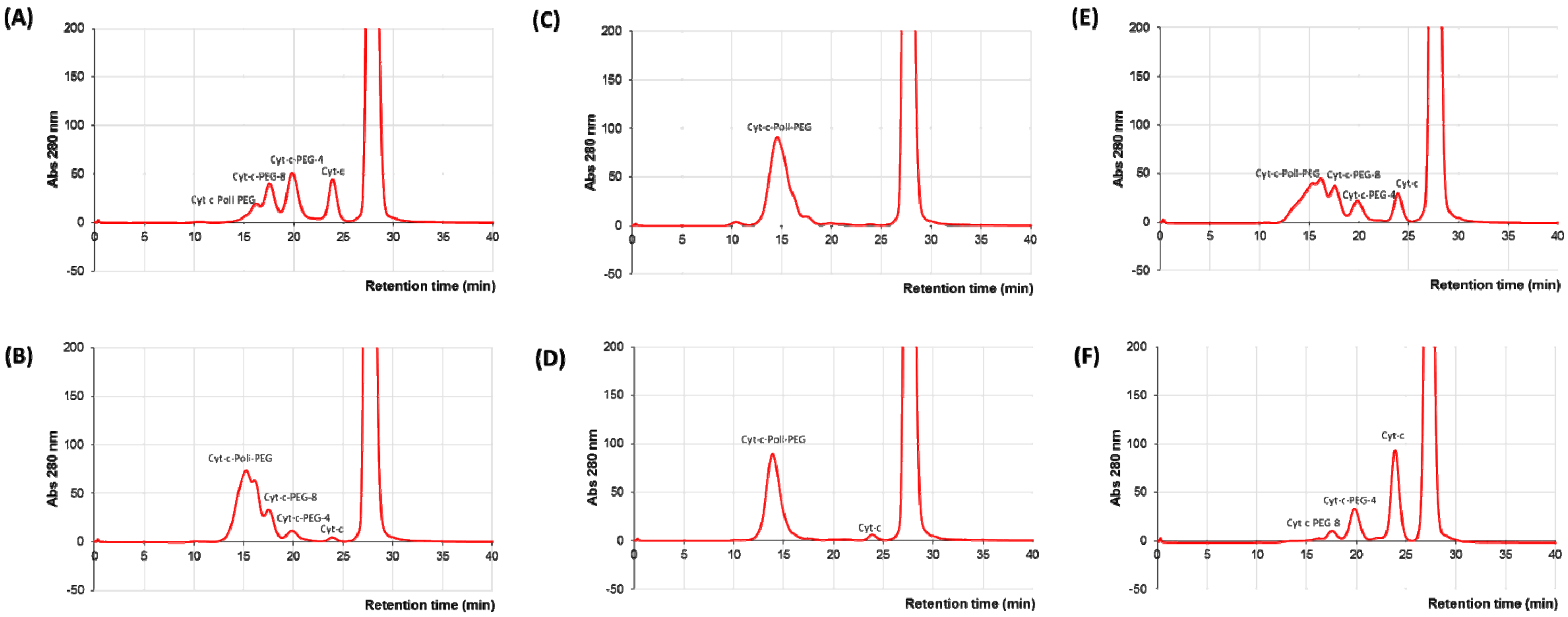

Figure S2. Effect of pH on cytochrome c (Cyt-c) PEGylation yield. Chromatogram of the PEGylation reaction mixture for the Cyt-c PEGylation in 0.1 M of potassium phosphate buffer at different pH values: 7 **(A)**, 8 **(B)**, 9 **(C)**, 10 **(D)**, 11 **(E)**, and 12 **(F)**. Reaction conditions: molar ratio 1:25 (protein:mPEG-NHS, 5 kDa), room temperature, 30 min. The peaks correspond to both Cyt-c PEGylated forms and unreacted protein.

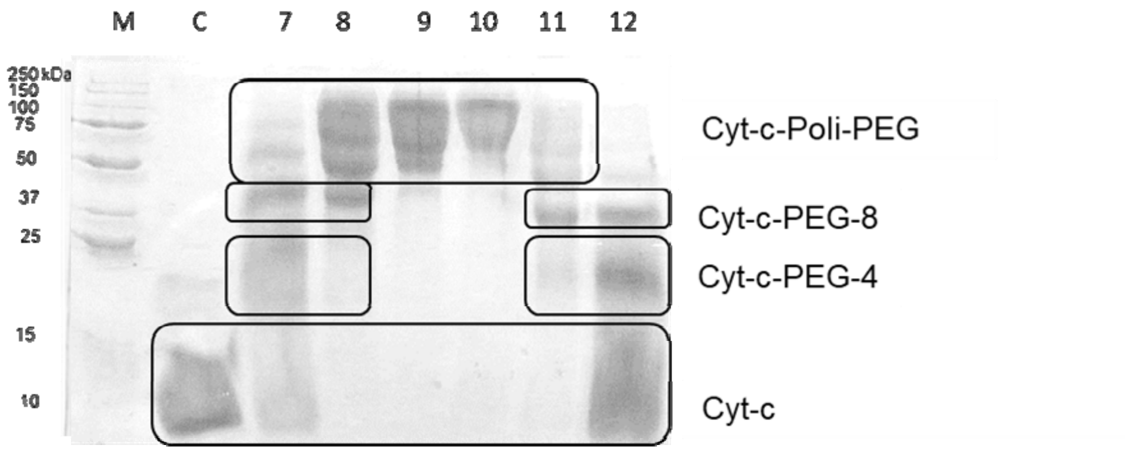

Figure S3. SDS-PAGE for the conjugation of mPEG-NHS to cytochrome c (Cyt-c) at different pHs. Lane 1: molecular weight marker, Lane 2: cytochrome c, Lane 3: pH 7, Lane 4: pH 8, Lane 5: pH 9; Lane 6: pH 10, Lane 7: pH 11, Lane 8: pH 12. The PEGylation reaction was performed in 0.1 M potassium phosphate buffer, 1:25 molar proportion (Cyt-c:mPEG-NHS, 5 kDa), at room temperature, for 30 minutes, and with a constant stirring of 400 rpm.

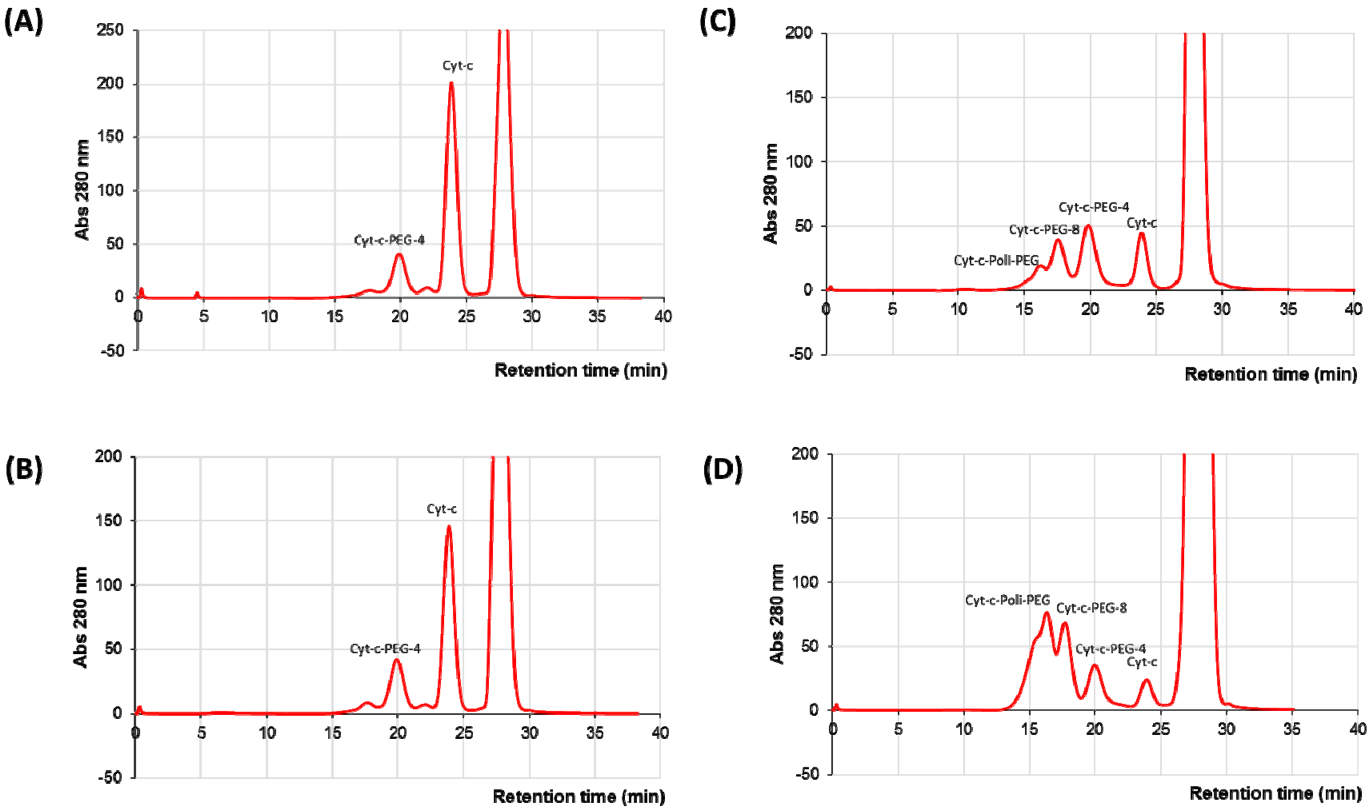

Figure S4. Effect of molar ratio (protein:mPEG-NHS) on cytochrome c (Cyt-c) PEGylation yield. Chromatogram of the PEGylation reaction mixture from a PEGylation reaction in 0.1 M phosphate buffer (pH =7) at different molar ratios (protein:mPEG-NHS, 5 kDa): 1:5 **(A)**, 1:10 **(B)**, 1:25 **(C)**, and 1:35 **(D)**, at room temperature for 30 minutes. The peaks correspond to both Cyt-c PEGylated forms and unreacted protein.

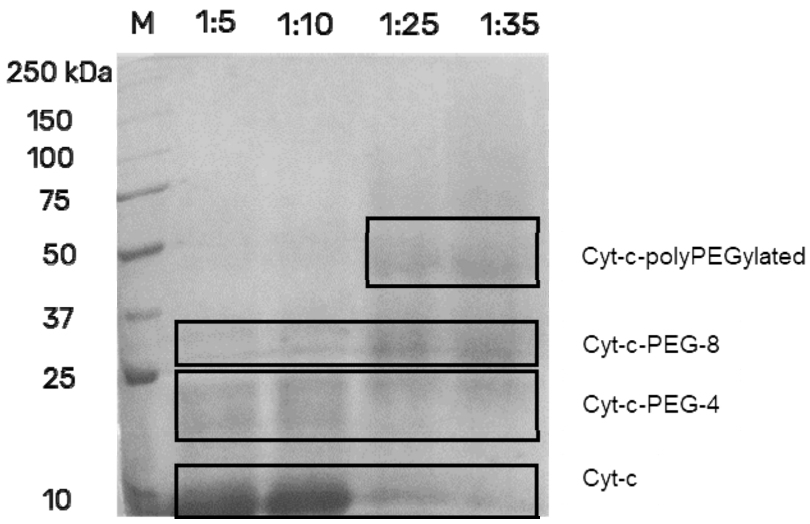

Figure S5. SDS-PAGE for the conjugation of mPEG-NHS with cytochrome c (Cyt-c) at different molar ratios (protein:mPEG-NHS). Lane 1: molecular weight marker, Lane 2: 1:5, Lane 3: 1:10, Lane 4: 1:25, Lane 5: 1:35. The PEGylation reaction was performed in 0.1 M potassium phosphate buffer (pH 7), at room temperature, for 30 minutes, and with a constant stirring of 400 rpm.

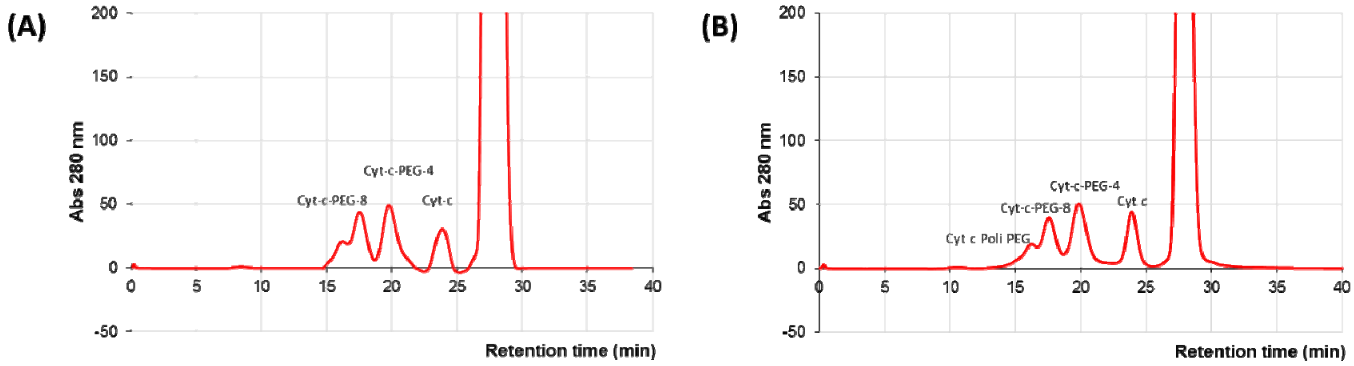

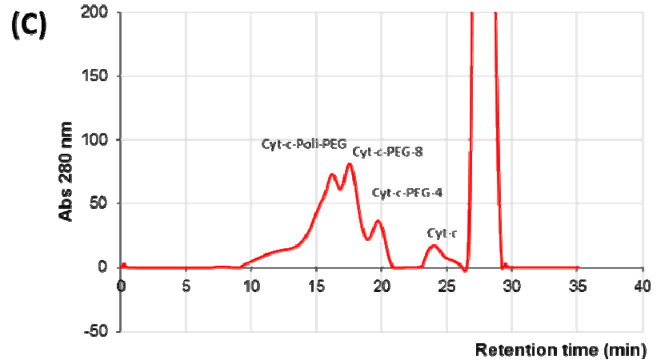

Figure S6. Effect of reaction time on cytochrome c (Cyt-c) PEGylation yield. Chromatogram of the PEGylation reaction mixture from a PEGylation reaction in 0.1 M phosphate buffer (pH 7), 1:25 molar proportion (protein:mPEG-NHS, 5 kDa), at room temperature for different reaction times: 15 (A), 30 (B), and 45 min (C). The peaks correspond to both Cyt-c PEGylated forms and unreacted protein.

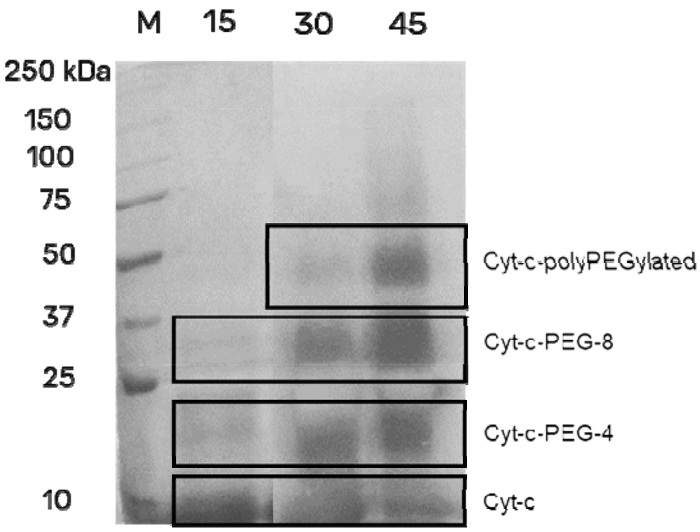

Figure S7. SDS-PAGE for the conjugation of mPEG-NHS with cytochrome c (Cyt-c) for different reaction times. Lane 1: molecular weight marker, Lane 2: 15 min, Lane 3: 30 min, Lane 4: 45 min. The PEGylation reaction was performed in 0.1 M potassium phosphate buffer (pH 7), 1:25 molar proportion (protein:mPEG-NHS, 5 kDa), at room temperature, and with a constant stirring of 400 rpm.

Table S1. Half-life ($t_{1/2}$) and long-term residual activity of native and PEGylated forms of cytochrome c (Cyt-c) stored in phosphate buffer at 4 °C and 25 °C.

**Cyt-c form**	**Half-life* (days)**		**Residual activity (%) on day 60**	
	**4 °C**	**25 °C**	**4 °C**	**25 °C**
Native	~44	~24	42.8	27.3
Cyt-c-PEG-4	>60	>60	62.2	53.4
Cyt-c-PEG-8	>60	>60	80.4	57.3

*Half-life (i.e. the time taken for the Cyt-c residual activity to be reduced by 50% of the initial) was estimated from data presented in Figure 6.
